# Observations in Electro-Therapeutics

**Published:** 1874-04

**Authors:** A. D. Rockwell

**Affiliations:** New York. Electro-Therapeutist to the New York State Women’s Hospital


ART. II.—*Observations in Electro-Therapeutics.* By A. D. ROCKWELL, M. D., of New York. Electro-Therapeutist to the New York State Women's Hospital.

In the light of our present knowledge of electro-physiology and its relations to electro-therapeutics, is is very certain that clinical experience, must in a great measure constitute the foundation upon which the latter is based. The recent researches in electro-physiology have, it is true, been sufficiently successful to establish a number of definite laws that are of practical value to therapeutics. Thus, we now know that the animal electricity of the body is variously modified under the use of the constant current according to the position of the poles, the direction of the current and the strength and length of the applications.

A correct appreciation of these various modifications enables us to treat with more intelligence and greater success, certain forms of neuralgia and paralysis, and many symptoms of central origin. We know that the effect of the application of electricity to the skin, depends on the kind of electricity used, its quantity, its tension, the character of electrodes employed &c, and we are enabled to confine the electrical influence to either the sentient or mortor nerves. In the treatment of anesthesia this fact must be considered.

It is found that direct galvanization of sympathetic nerve exercises an immediate and powerful influence over the circulation in the arterioles, and in therapeutics it is possible, up to a certain point to utilize this discovery.

The elaborate experiments of Brenner, show that the auditory nerve reacts to the galvanic current by certain fixed laws, and it is claimed that the practical advantage of an acquaintance with these laws consists in this, that any deviation from them, must be regarded as abnormal, and indicate a pathological condition. These and many other electro-physiological facts sufficiently attest the importance of conjointly studying electricity in its physiological and therapeutical relations; but until the science of electro-physiology has advanced far beyond its present status, it will never do to make it the sole, or even the chief foundation for electro-therapeutics. It is therefore unwise to refuse, as have some, to accept a clinical fact, because in their judgment it does not seem perfectly adjusted in its relations to known physiological laws. Such persons forget that there are laws in electro-physiology yet to be known, that in all departments of science, facts are observed long before a solution is found for them, and that here, as elsewhere, we should no more hesitate to accept well authenticated therapeutical results because an explanation is not readily found in physiology, than we should hesitate to accept any truth of science in general, because its satisfactory explanation is not immediately at hand.

The methods employed in most of the following cases it will be observed, were either general faradization or central galvanization or an alternation of the two. I have in detail and on various occasions described these methods, and hence, even if space permitted it, would hardly be necessary to enter into a full explanation here.

It is sufficient to say that the effects of a thorough and well directed application of general faradization have an equally wide range, (paradoxical as it may seem,) as a stimulating tonic, or as a direct sedative. In regard to central galvanization, its power to directly effect the brain—the possibility of localizing it in special parts of the brain—the liability of its use leading to serious central disturbance and its value as a remedial method much has been written both pro and con. So far as concerns its direct action on the brain, no fact in physiology is more firmly established. That it is impossible to localize it in very circumscribed portions of the brain must be acknowledged, but that if used with the requisite caution and skill, there is any danger either near or remote has been most thoroughly disproved, and lastly, the assertion that it is useless as a remedial method must be proved or disproved by the teachings of actual and varied experience. The results of treatment by electrization here recorded, although not to be considered typical of what can be obtained in all cases presenting the same apparent indications, yet serve to illustrate the *possibilities* of electro-therapeutics.

### *Remarkable Effects of general Faradization in a case of Acute Mania.*

In the following case, although no permanent relief was afforded by the method employed, the temporary effects were so sudden and starling as to render the history of exceeding interest. The patient, a Miss R., aged 20, living in Harlem, and under the care of Dr. Joseph Worster, of N. Y., was suffering from acute mania, dating from suppression of the menses that occurred four months before she fell under my care. She had always enjoyed most excellent health, indeed, was remarkable for her vigorous, robust constitution. While watering the plants in the conservatory of her sister, her clothes became quite wet; she neglected to change them immediately, and the consequence was a suppression of the menstrual flow. She complained on the following day of severe headache, and on occasions during the next two weeks was markedly unreasonable in her actions and demands. Finally active delirium set in, but with no decrease of bodily strength. At times she was intensely violent in her demonstrations, screaming at the top of her voice and breaking every article of furniture within her reach; as a consequence she was confined in a room striped of its furniture, and in her wildest moods the straight jacket was applied. For more than two months no sleep visited her eyelids without the nightly administration of from 100 to 120 grs. of chloral. During the morning she was often measurably quiet, but as evening approached, she became absolutely ungovernable, and when chloral was not given, she had been known to pace around the room with great rapidity and strength, muttering to herself, with absolutely no cessation from sunset to sunrise. She had decreased in weight from 160 to 110 lbs. On the evening of the 15th of April, she was held firmly in position by several assistants, and after moistening the hair of the head, I submitted her to the most thorough form of general faradization, with the very finest current obtainable. The current was of great strength, but in itself, evidently caused no discomfort to the patient. That night and without the use of any drug, the patient enjoyed five hours of the quietest sleep, and for forty-eight hours thereafter she was perfectly tractable. Another paroxysm of violence again showed itself, and the same form of application was re-administered. She once more slept quietly, and in the morning awoke quite rational but exceedingly weak in body. The day being warm and bright, a chair was placed in the yard where she sat for several hours, and in all her conversation evinced entire freedom from any thing like mental derangement. Suddenly however she arose and ran around the yard with great rapidity. She was immediately captured, and when taken to her room, gave evidence of all her former derangement. She was very violent during the rest of the day, but after the administration of 100 grs. of chloral, she passed the latter part of the night in comparatively quiet sleep. On the following morning she awoke somewhat excited, and so remained during the day, while towards evening she as usual became more violent. General faradization was again tried and was attended by its previous good effects. Four or five hours of quiet sleep followed, and on awakening and for a part of the succeeding day the patient was quite calm, and in some respects entirely rational. Not to prolong the case, it may be said that this same method was repeated several times, and on each occasion was followed by sleep, and on awakening, by a calmer mind. More than this, during my opportunity for investigaticn, it failed to accomplish; and as the patient was at this time taken to an asylum by her friends, she passed from under my observation.

It has been suggested, that in the above case, the calm sleep that followed the treatment was due more to the exhaustion occasioned by the struggles of the patient, than to the effects of the faradization. This theory is hardly worthy of acceptance, since the struggles when the chloral was administered, were as violent and sometimes as long continued, and it was not only evident that sleep was of shorter duration, and less profound, but that there was an utter absence of those lucid and peaceful hours, that were so manifestly the result of electrization. It is to be regretted that it was impracticable to retain the patient longer under observation, or to attempt central galvanization, which has proved so undeniably of value in melancholia and kindred conditions.

It would have been desirable as well in this case, to have made use of intra vaginal or even intra-uterine galvanization for the purpose of exciting menstruation.

*Failure of the intellectual powers, accompanied by impairment of the faculties of special sense, perverted sensations in the extremities, together with paraplegic symptoms. Approximate recovery under general faradization and central galvanization.*

Mr. M., an actor of 20 years standing, was placed under my care by Dr. F. L. Harris, of N. Y. The patient was a temperate man, and so far as his profession permitted he was regular in all his habits; but the character of his engagements had rendered it necessary for him to exercise his memory through a series of years to an unusual and as the sequel proved, to a most injurious extent.

Two months prior he began to observe that his intellectual powers were failing him. His memory became so impaired, and his thoughts so confused that he found it utterly impossible to "commit" anything new, or recall readily certain "parts" that had been long perfectly familiar. At the same time his limbs became weak, and he complained of sensory symptoms in the tops of the fingers, much the same as those present after frost bite.

The integrity of most of the senses was markedly impaired. The sight especially had failed him to such a degree, that it was with difficulty that he could read at all. The patient was exceedingly timid, and had a nervous dread of the treatment proposed, but he was at once submitted to a gentle but most thorough seance of general faradization, which was followed by an immediate improvement in the power of locomotion. After a day's interval central galvanization was employed, and this alternate treatment was continued for a month. The annoying sensory symptoms disappeared, he gained entire mastery over his limbs, his strength of vision became normal, and when I last saw him there had been sufficient improvement in his intellectual faculties to enable him successfully to attempt a performance on the stage.

*Throat Dysæsthesia associated with severe Neuralgic Headaches of twenty years standing. Approximate recovery under Central Galvanization.*

Mrs. T., who was directed to me by Dr. C. R. Agnew, had for twenty years suffered from an almost constant and painful heaviness about the head and eyelids. Associated with this symptom were frequent periodical attacks of intense cephalalgia.

For the last few years, the patient had complained of a local neuralgia of somewhat rare occurrence, noticed by Hanfield Jones, as "throat dysæsthesia." Dysphagia was present with a sense of impending suffocation, and with heat and dryness of the mucus membrane.

Inspection revealed no inflammation sufficient to account for the distress. The treatment consisted of some twenty-five applications, and almost wholly by the method of central galvanization, and with the most decidedly beneficial results. The heaviness of the head and eyes were greatly relieved, and the cephalalgia occurred at far greater intervals, and with no approach to the former severity. The throat difficulty yielded more readily and completely than the other symptoms.

*Two cases of long standing Paralysis of the Œsophagus. Immediate recovery under localized galvanization.*

Mr. P., aged 45, was directed to me by Dr. James Anderson, of New York, with symptoms that at first sight suggested atrophy of the motor roots of the upper spinal nerves. A more careful study of the case rendered it evident that the patient was not suffering from glosso-pharyngeal paralysis, but rather from paralysis and occasional spasms of the œsophagus. The patient had thus suffered more or less for three years, and for the last year the distress had been constant. It was impossible for him to swallow without a sense of impending suffocation, and at all times there was a feeling as if the tube was drawn into a knot. Almost every conceivable remedy had in vain been tried with the exception of electricity. I submitted him to localized galvanization, and after the third application, almost every symptom disappeared, and in a couple of weeks he had quite recovered. More than a year has passed, but as yet there has been no relapse.

In a case somewhat similar referred to me by Dr. Fordyce Barker, like immediate success followed substantially the same treatment,

*A case simulating Palsy Agitans, and associated with spasmodic muscular contractions and neuralgic pains. Approximate relief from central galvanization and general faradization.*

Mrs. M., aged 50, applied to me for the relief of a disorder, of which the following were the main symptoms. These symptoms had been almost constant for more than fifteen months. The patient was chlorotic, and so exceedingly feeble, that a walk of a few blocks, caused complete exhaustion. There were severe neuralgic pains in the face, right arm and along the spine, but no tenderness to pressure in the latter situation.

The most annoying symptoms were frequent spasmodic contractions of the muscles of the neck, while a constant and incessant trembling of the hands during the waking hours, made with the rest a complication of symptoms that suggested possible structural change of the upper portion of the cord.

Central galvanization was in this case alternated with general faradization. The effect was an immediate and complete relief of the spasmodic muscular contraction. The neuralgia was gradually dispelled, and the trembling or shaking was so benefitted in the course of two months' treatment as to be hardly noticable. During the summer that followed, the patient was almost entirely free from every unpleasant symptom.

*Amenorrhœa in a woman aged 49, associated with partial paralysis and other symptoms. After two seances the menses re-appear followed by marked general improvement.*

Mary M., stated that three years since, although at that time she was 46 years of age, her menses suddenly stopped, leaving her in a condition of partial paralysis, anæsthesia, and periodical attacks of distressing fullness and oppression about the head. Because of her age I did not think to restore the courses, but on general principles, submitted her to general faradization.

After two applications the menses re-appeared and lasted a day and a half. She was immediately much improved in all her symptoms. The anesthesia almost entirely disappeared, she was able to walk with ease and firmness, and the head symptoms ceased to distress her. With the subsequent history of the case I am not familiar.

*Violent hysterical symptoms dependent on suppressed menstruation alleviated by two seances of general faradization and local galvanization.*

A most violent persistent case of hysteria in the person of a married lady, aged 40, came under my observation through the kindness of Dr. Oliver White. The patient was in bed—suffering from violent paroxyms of alternate weeping and screaming. The hands and feet were cold, the pulse feeble, and the pain in the head was constant and of the most severe character.

These symptoms had continued for more than forty-eight hours, and in order to evert serious consequences, it seemed as if in some way relief must soon be afforded.

The menstrual period was delayed nearly two weeks, and to this circumstance it was possible in part to attribute the attack. The patient was submitted to general faradization, and immediately after a galvanic current from eight cells was as nearly as possible localized in the uterus. These efforts were followed by a decided alleviation of the symptoms, and a tolerably quiet night was the result. The menses did not however appear, and on the following night I again gave the same treatment, slightly increasing the tension of the galvanic current. Before morning the menstrual flow appeared, and there was no further evidence of nervous disturbance Nearly a year subsequently, the patient experienced another attack of like character, and substantially the same treatment again relieved her.

*Choreic disturbance of the head of five months standing. Recovery under twelve applications of general faradization.*

Minnie V., aged 8 years, had been afflicted for five months with severe and almost constant nervous twitchings of the head. They were evidently choreic in character, occurred without the consciousness of the child, but during sleep were entirely absent. The patient was somewhat depressed in health and decidedly anemic, and I therefore submitted her to general faradization.

Under the influence of a dozen applications, she gained in appetite and strength. The choreic disturbance became very greatly improved, and after the cessation of the treatment for the purpose of allowing the *secondary effects* to be manifested; it was not more than two weeks before the recovery was complete. No relapse has occurred.

In some and perhaps the majority of the above cases, the results cannot be explained fully and satisfactorily by electro-physiology, and yet these results do not lack for comformation. Electro-physiology is constantly being extended, so that we are becoming more and more able to explain the therapeutics of electricity, and it is not improbable that the near future may enable us to offer a scientific solution for most of the electro-therapeutical results of the present day.

Meanwhile, it is certainly proper to extend our practical experience, so far as possible, even if we are at all times thus kept a little in advance of electro-physiology.

